# Supplementary material for: INSTIs and NNRTIs Potently Inhibit HIV-1 Polypurine Tract Mutants in a Single Round Infection Assay
Source: Viruses. 2021 Dec 14;13(12):2501. doi: 10.3390/v13122501 (PMC8705849; doi:10.3390/v13122501)
Supplement: Supplementary file 1 [file viruses-13-02501-s001.zip › SUPPLEMENTALINFORMATIONPPT mutant study.pdf]

Table S1. Comparison of antiviral potencies of INSTIs against a panel of HIV-1 PPT mutants.

	WT	AGC	AGT	+G
**BIC**	0.48 ± 0.09 nM	0.49 ± 0.07 nM	0.49 ± 0.06 nM	0.55 ± 0.10 nM
**DTG**	1.1 ± 0.1 nM	0.86 ± 0.06 nM	0.67 ± 0.08 nM	0.77 ± 0.06 nM
**CAB**	0.9 ± 0.02 nM	0.79 ± 0.1 nM	2.4 ± 0.3 nM	0.66 ± 0.04 nM
**RAL**	3.0 ± 0.4 nM	1.5 ± 0.3 nM	3.0 ± 0.5 nM	2.1 ± 0.3 nM
**EVG**	1.1 ± 0.3 nM	0.52 ± 0.06 nM	0.51 ± 0.04 nM	0.53 ± 0.05 nM
**4c**	3.8 ± 0.3 nM	2.4 ± 0.5 nM	3.2 ± 0.4 nM	2.8 ± 0.5 nM
**4d**	0.67 ± 0.09 nM	0.5 ± 0.05 nM	0.6 ± 0.1 nM	0.58 ± 0.09 nM
**4f**	0.91 ± 0.11 nM	0.9 ± 0.1 nM	0.69 ± 0.05 nM	0.78 ± 0.07 nM
**6b**	0.53 ± 0.02 nM	0.41 ± 0.04 nM	0.53 ± 0.18 nM	0.47 ± 0.08 nM
**6v**	195.0 ± 27.2 nM	85.4 ± 7.5 nM	60.5 ± 3.8 nM	123.9 ± 3.3 nM

Table S1. Comparison of antiviral potencies of INSTIs against a panel of HIV-1 PPT mutants. The EC_{50} values and the standard deviation (n=3) of the FDA-approved INSTIs BIC, DTG, CAB, RAL, and EVG and our compounds 4c, 4d, 4f, 6b, and 6v against WT HIV and the HIV-1 PPT mutants are shown.

Table S2. Comparison of antiviral potencies of NNRTIs against a panel of HIV-1 PPT mutants.

	WT	AGC	AGT	+G
**EFV**	0.1 ± 0.01 nM	0.11 ± 0.02 nM	0.1 ± 0.01 nM	0.12 ± 0.01 nM
**RPV**	0.15 ± 0.02 nM	0.14 ± 0.03 nM	0.14 ± 0.03 nM	0.2 ± 0.02 nM
**DOR**	0.34 ± 0.04 nM	0.3 ± 0.03 nM	0.27 ± 0.02 nM	0.36 ± 0.02 nM
**6**	0.15 ± 0.03 nM	0.12 ± 0.01 nM	0.14 ± 0.02 nM	0.16 ± 0.02 nM
**7**	0.15 ± 0.01 nM	0.18 ± 0.02 nM	0.19 ± 0.02 nM	0.17 ± 0.01 nM
**11**	0.15 ± 0.01 nM	0.17 ± 0.03 nM	0.19 ± 0.02 nM	0.17 ± 0.02 nM
**12**	0.3 ± 0.04 nM	0.39 ± 0.03 nM	0.26 ± 0.05 nM	0.39 ± 0.06 nM
**13**	0.21 ± 0.03 nM	0.26 ± 0.02 nM	0.14 ± 0.02 nM	0.25 ± 0.02 nM
**16**	0.1 ± 0.0 nM	0.05 ± 0.01 nM	0.08 ± 0.01 nM	0.09 ± 0.01 nM
**27**	0.15 ± 0.03 nM	0.12 ± 0.01 nM	0.11 ± 0.01 nM	0.16 ± 0.01 nM

Table S2. Comparison of antiviral potencies of NNRTIs against a panel of HIV-1 PPT mutants. The EC_{50} values and standard deviation (n=3) of the FDA-approved NNRTIs EFV, RPV, and DOR and our compounds 6, 7, 11, 12, 13, 16, and 27 against WT HIV and a panel of HIV-1 PPT mutants are shown.
